# Supplementary figures and images for: 2′,3′ cyclic nucleotide 3′ phosphodiesterase 1 functional isoform antagonizes HIV-1 particle assembly
Source: Life Sci Alliance. 2024 Jan 2;7(3):e202302188. doi: 10.26508/lsa.202302188 (PMC10761555; doi:10.26508/lsa.202302188)

Fig 1

C

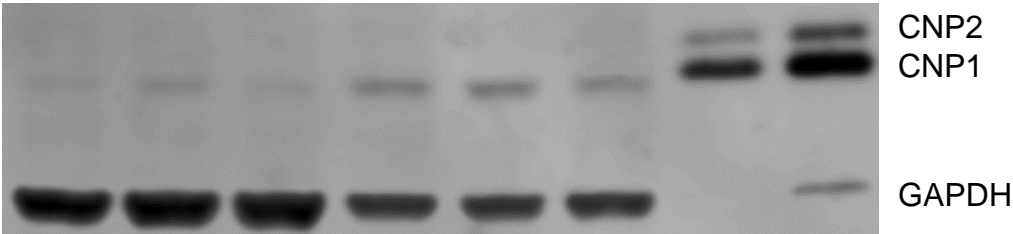

D

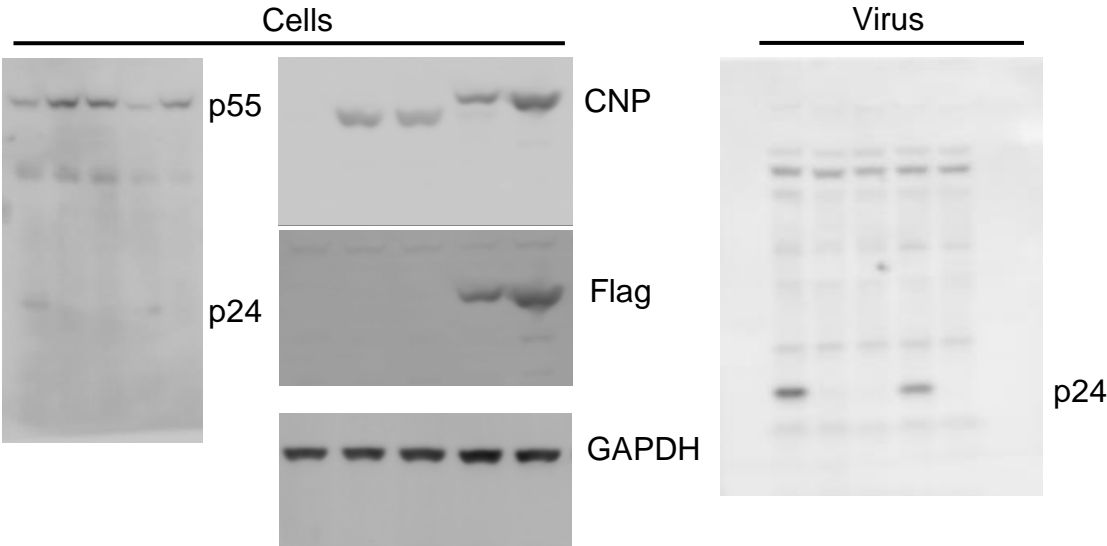

F

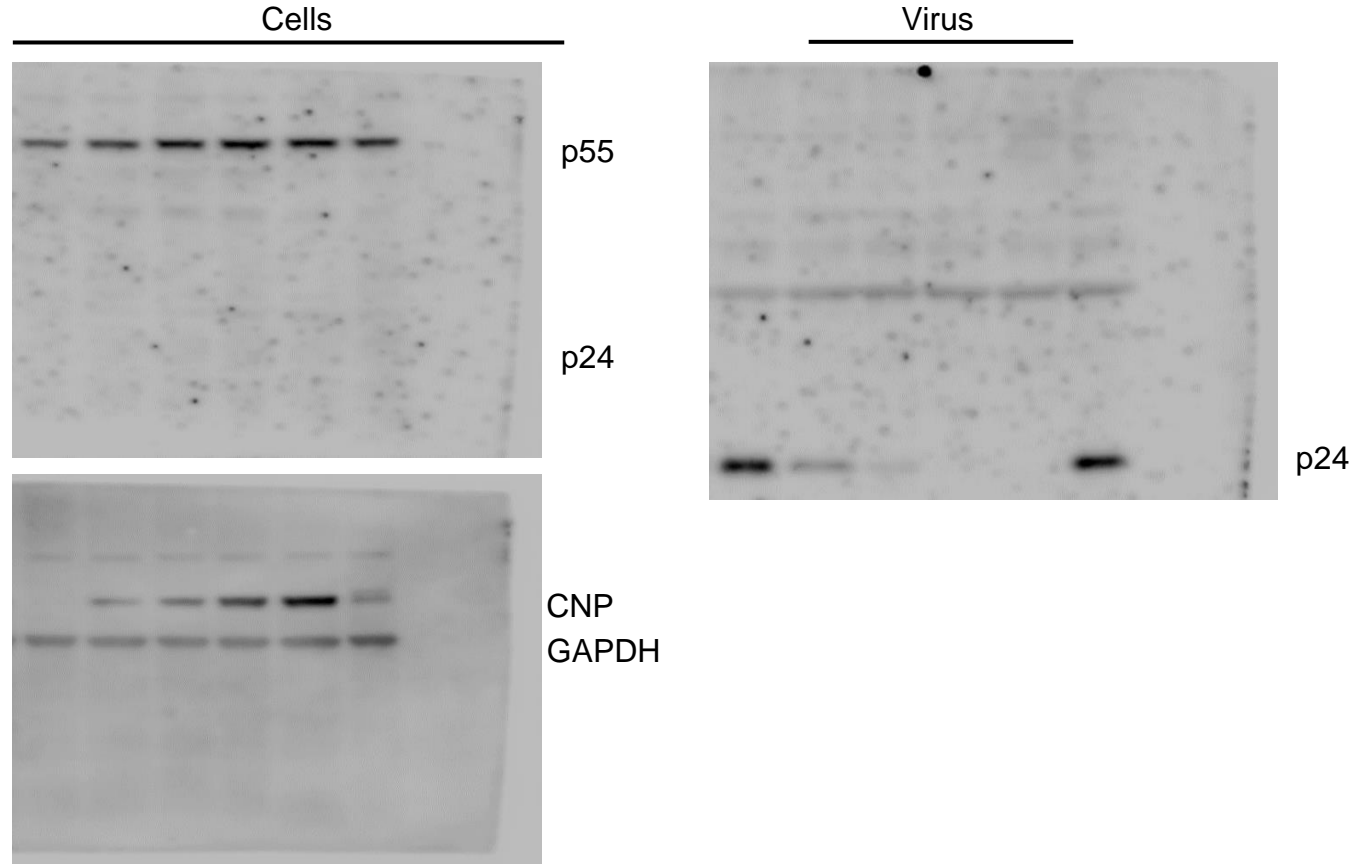

Fig 2

A

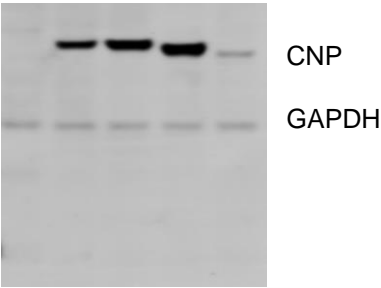

B

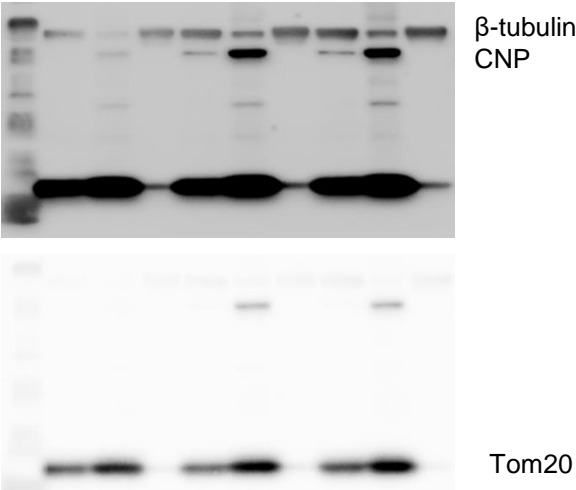

E

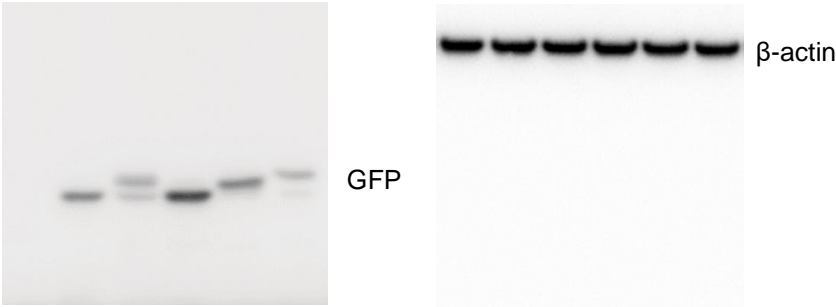

F

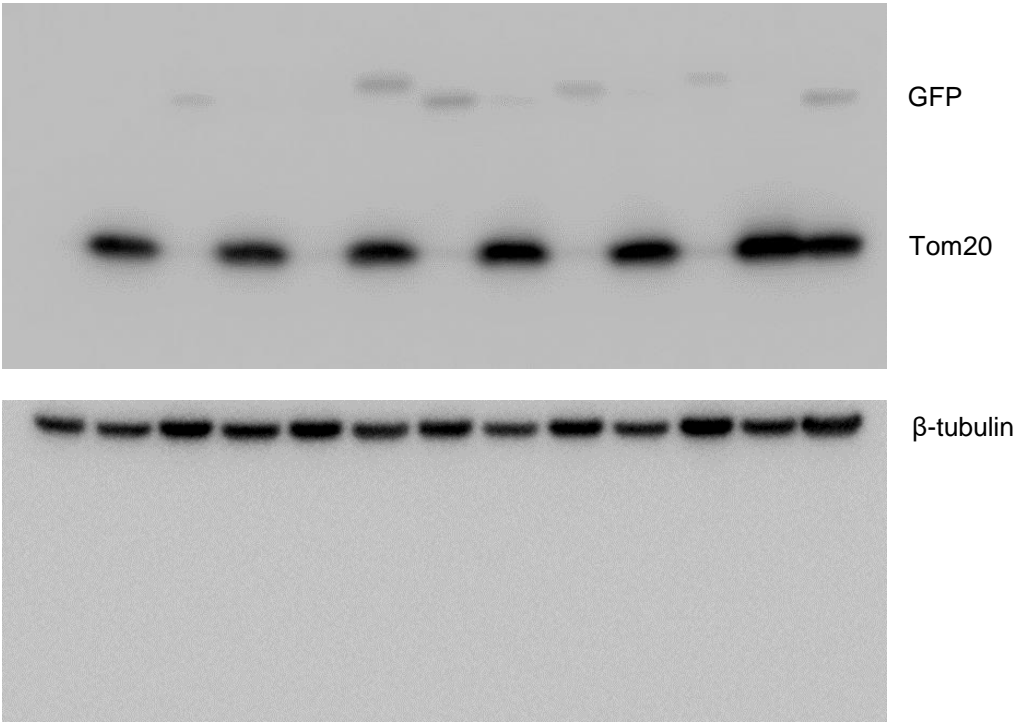

Fig 3

A

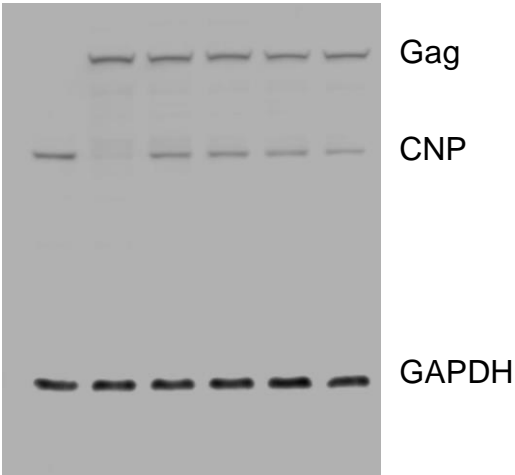

G

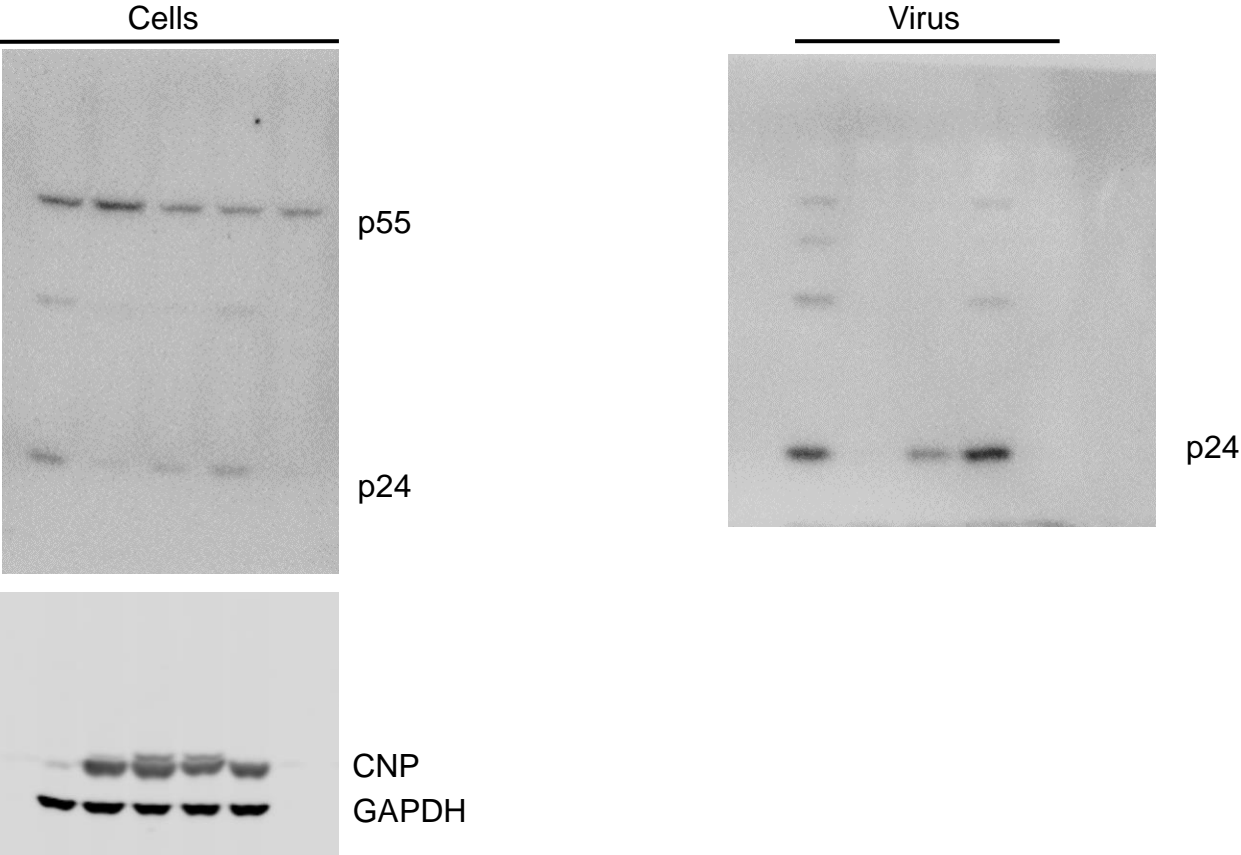

Fig S2B

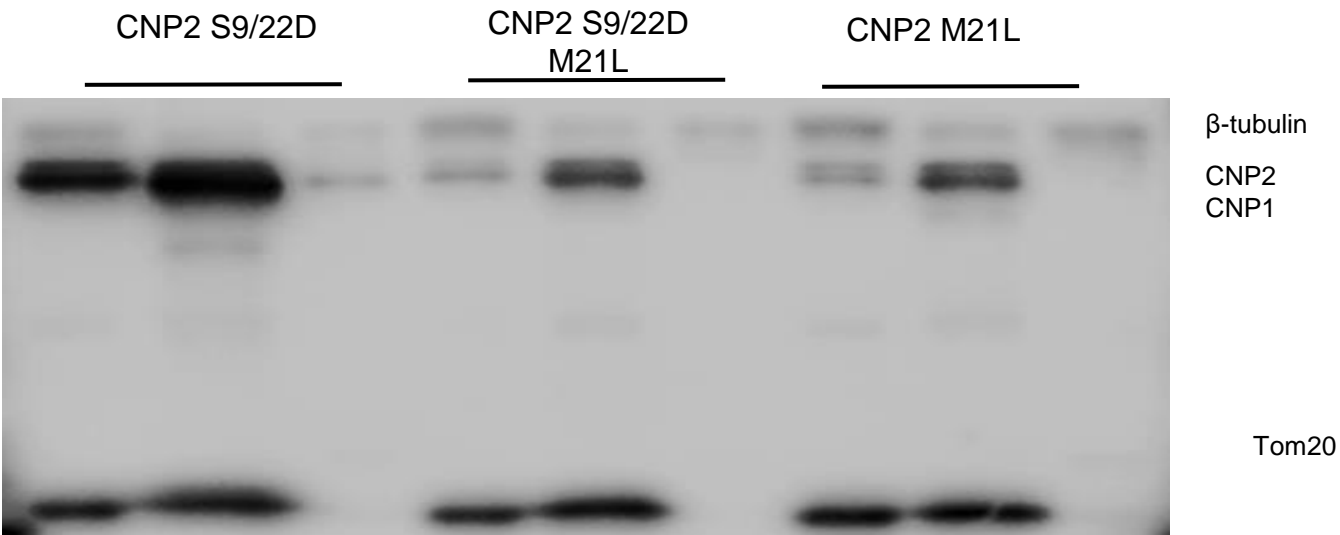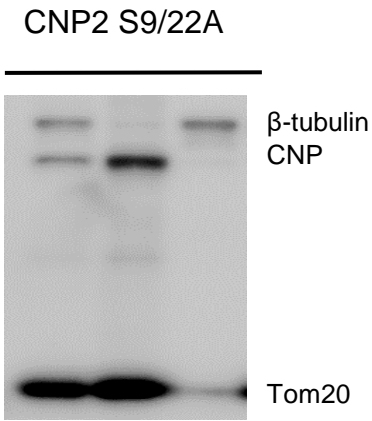

Fig S3B and C

**B**

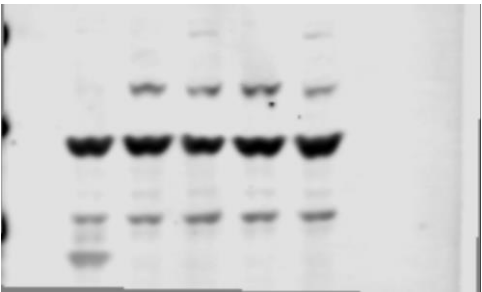

**C**

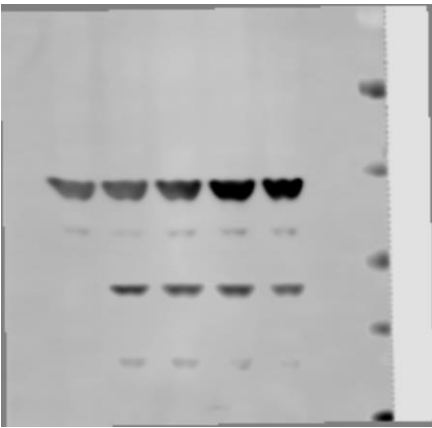

Fig S4E

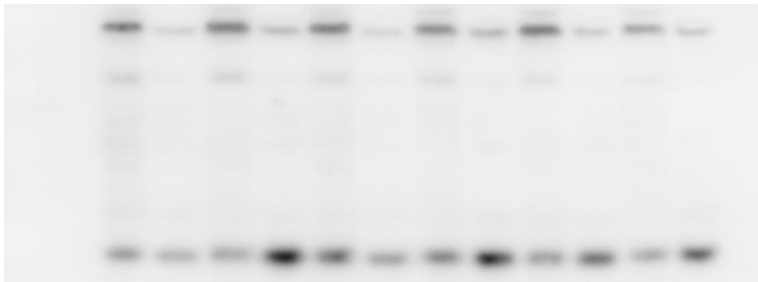

Fig S5B

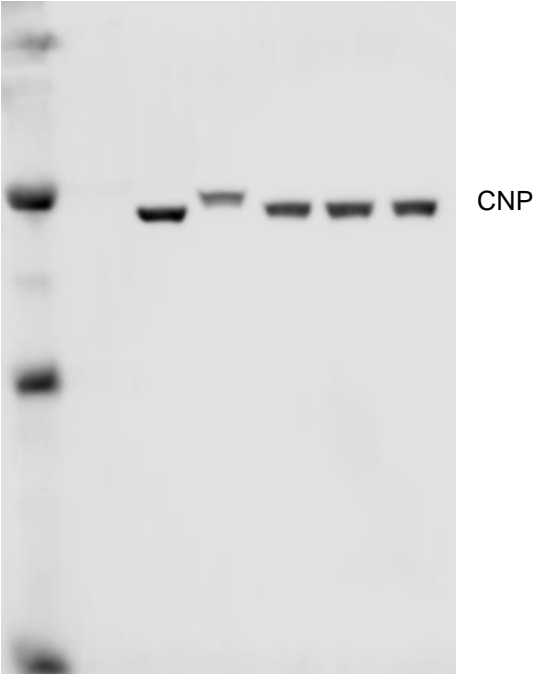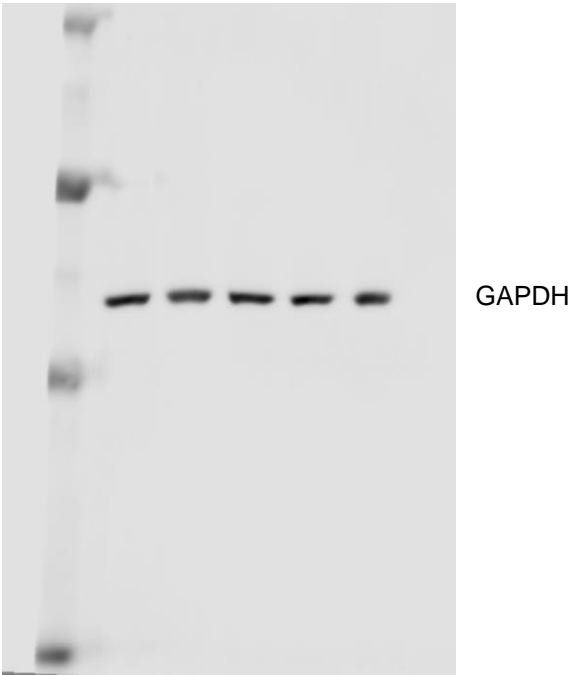

Supplement: Supplementary file 1 [file LSA-2023-02188_SdataF1_F2_F3_FS2_FS3_FS4_FS5.pdf]
